# Titanium Dioxide (TiO_2_) Nanoparticles Preferentially Induce Cell Death in Transformed Cells in a Bak/Bax-Independent Fashion

**DOI:** 10.1371/journal.pone.0050607

**Published:** 2012-11-21

**Authors:** Yanglong Zhu, John W. Eaton, Chi Li

**Affiliations:** Molecular Targets Program, James Graham Brown Cancer Center, Department of Medicine, Department of Pharmacology and Toxicology, University of Louisville, Louisville, Kentucky, United States of America; Roswell Park Cancer Institute, United States of America

## Abstract

While the cytotoxic effects of titanium dioxide (TiO_2_) nanoparticles have been under intense investigation, the molecular mechanisms of this cytotoxicity remain unknown. Here we investigated the influence of oncogenic transformation and a major apoptotic signaling pathway on cellular responses to TiO_2_ nanoparticles. Isogenic wild-type (WT) and apoptosis-resistant (Bak^−/−^Bax^−/−^) cell lines with and without tumorigenic transformation were examined. TiO_2_ nanoparticles preferentially reduced viability of tumorigenic cells in a dose-dependent fashion compared with their untransformed counterparts. Importantly, the elevated cytotoxicity of TiO_2_ nanoparticles was independent of a major Bak/Bax-dependent apoptosis pathway. Because transformation does not affect cellular fluid-phase endocytosis or nanoparticle uptake, it is likely that the increased cytotoxicity in tumor cells is due to the interaction between TiO_2_ nanoparticles and the lysosomal compartment. Overall, our data indicate that TiO_2_ nanoparticles induce cytotoxicity preferentially in transformed cells independent of a major apoptotic signaling pathway.

## Introduction

Titanium dioxide (TiO_2_) nanoparticles have a wide range of commercial applications, particularly in consumer products [1]. Emerging evidence suggests that the unique physical and chemical properties of TiO_2_ nanoparticles, such as ultra-small size, increased surface area per unit mass, chemical composition, surface structure, shape, aggregation, and high reactivity, may pose potential risks to human health and the environment [2], [3]. Through inhalation, ingestion, and injection, TiO_2_ nanoparticles can enter the human body, where they may interact with cells and components of cells, such as proteins and lipids, to compromise cellular functions, leading to cell toxicity [4]–[6]. Research on animal models has further confirmed the cytotoxic effects of TiO_2_ nanoparticles. For instance, intratracheal instillation of TiO_2_ nanoparticles in mice causes pulmonary inflammation, emphysema, and epithelial cell apoptosis [7]. Similarly, oral delivery of TiO_2_ nanoparticles leads to inflammation and damage to liver and kidney [8]. The cytotoxic effects of TiO_2_ nanoparticles depend on physicochemical properties of TiO_2_ nanoparticles, particularly their size, with smaller particles causing more damage than bigger ones [9], [10].

*In vitro* experiments involving the effects of TiO_2_ nanoparticles on various cell lines have generally confirmed the results obtained from animal studies. However, conflicting observations have made it difficult to establish a detailed molecular mechanism of TiO_2_ nanoparticle cytotoxicity. In many studies, upon TiO_2_ nanoparticle exposure, damage to lipids, proteins, and DNA leads to damage of subcellular organelles and cell death [11]–[15]. While TiO_2_ nanoparticles have been reported to induce chromatin condensation, nuclear fragmentation, caspase activation, and ultimately apoptosis [11], [14], [15], cells treated with TiO_2_ nanoparticles can also exhibit the features of non-apoptotic (e.g., necrotic) cell death, such as cytoplasmic membrane rupture [12], [13], [16]. Furthermore, there are even reports of cells that are resistant to TiO_2_ nanoparticle toxicity [17]. Recent studies provide evidence that the physicochemical properties of TiO_2_ nanoparticles and types of cells studied determine the cytotoxic activities of TiO_2_ nanoparticles [10], [16]. A common feature emerging from these studies is that exposure of cells to TiO_2_ nanoparticles increases the generation of reactive oxygen species (ROS) [18]–[21]. However, whether or not the increase of ROS is truly responsible for the cytotoxic effects of nanoparticles is still unknown.

In multicellular organisms, cell death is involved in many physiological and pathological processes [22], [23]. Cell death is a highly heterogeneous process in which several distinct, in some cases partially overlapping, cell signaling cascades can be activated and display different morphological characteristics. Apoptosis, a tightly controlled cellular suicide program, is a major mode of cell death, and is regulated by the Bcl-2 family of proteins [24]–[26]. All Bcl-2 proteins share one or more distinct domains of homology named the Bcl-2 homology (BH) domains to promote or inhibit apoptosis. Bak and Bax are redundant multi-domain pro-apoptotic Bcl-2 proteins, and cells deficient in both proteins are unable to undergo apoptosis in most apoptotic paradigms, indicating that Bak/Bax mediate major apoptotic signaling responses [27], [28].

TiO_2_ nanoparticles have been shown to be able to induce tumor cell death [16]. How apoptotic signaling pathways might be involved in tumor cell death caused by TiO_2_ nanoparticles is unclear. To explore how oncogenic transformation affects the cytotoxicity of TiO_2_ nanoparticles, we examined mouse embryonic fibroblasts (MEF) transformed with the oncogene K-Ras and the DNA tumor viral oncogene E1A as well as their isogenic untransformed counterparts. E1A cooperates with K-Ras to transform primary fibroblasts by suppressing p53 activities and abrogating K-Ras-induced cellular senescence [29], [30]. As the development of fibrosarcoma is associated with K-Ras oncogene and inhibition of p53 signaling [31]–[33], the transformed cell lines examined in our studies are likely representative of fibrosarcoma. Here, we report that TiO_2_ nanoparticles preferentially induce cell death in transformed cells compared with their untransformed counterparts. Importantly, the cytotoxicity of TiO_2_ nanoparticles is independent of Bak/Bax-mediated apoptosis. We also find that the selective cytotoxicity of TiO_2_ nanoparticles might be related to an increase of lysosomal activities induced by oncogenic transformation. Overall, our results may suggest strategies for using TiO_2_ and other types of nanoparticles as new anti-cancer agents.

## Results

### Oncogenic Transformation does not Influence Bak/Bax-dependent Apoptosis

**Figure 1:**
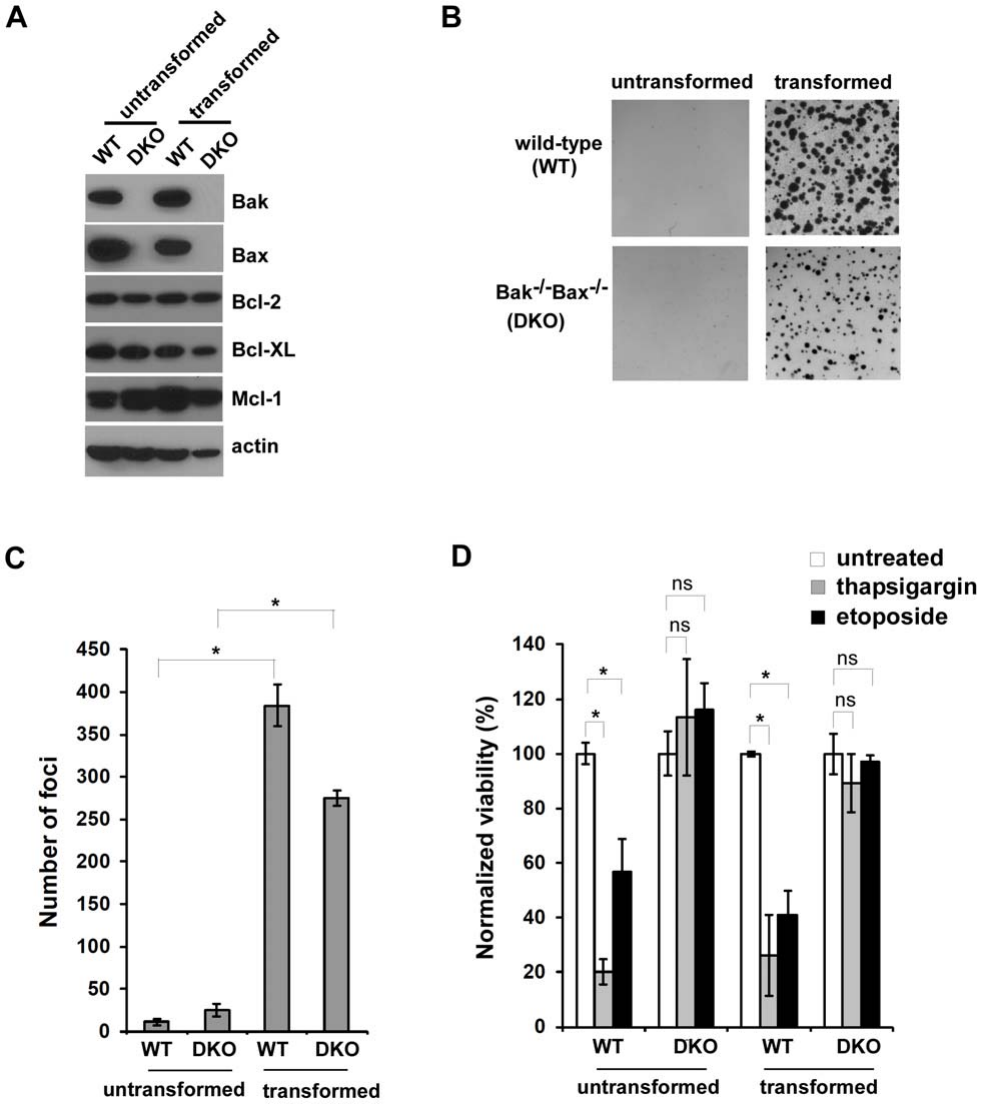
Bak and Bax mediate apoptosis in Ras/E1A-transformed MEF cells. (**A**) Expression of both anti-apoptotic and pro-apoptotic Bcl-2 proteins in the indicated cell lines were examined by western blot. (**B**) The indicated MEF cells were cultured in soft agar and representative images are shown. (**C**) The number of foci shown in (B). Data represent mean±S.D. of three independent measurements. Asterisks (*) indicate *P*<0.05, Student’s unpaired t test. (**D**) Both untransformed and transformed Bak^−/−^Bax^−/−^ MEF cells were resistant to apoptotic stimuli. The indicated cells were treated with 0.5 µM thapsigargin and 5 µM etoposide, and cell viability was measured 48 hours later. Mean±S.D. of three independent experiments are shown. Asterisks (*) indicate *P*<0.05, “ns” indicates no significance (P>0.05), Student’s unpaired t test.

Previous studies have demonstrated that titanium dioxide (TiO_2_) nanoparticles are cytotoxic to both tumor and nontumor cells, although detailed molecular mechanisms are still unknown [16]. To systematically investigate how transformation and apoptotic signaling pathways are involved in TiO_2_ nanoparticle-induced cytotoxicity, we studied two pairs of untransformed mouse embryonic fibroblasts (MEFs) and their isogenic counterparts transformed by expression of the oncogenes K-Ras and E1A: one pair of cells expressed all Bcl-2 proteins (wild-type, WT) and another pair was deficient in expression of two key proapoptotic Bcl-2 proteins, Bak and Bax (Bak^−/−^Bax^−/−^) (Figure 1A). Soft agar colony formation assays indicated that both transformed WT and Bak^−/−^Bax^−/−^ cells were able to proliferate in an anchorage-independent fashion but untransformed cells did not form colonies (Figures 1B and 1C). To examine whether oncogenic transformation affects cellular responses to apoptotic stimuli, transformed and untransformed cells were treated with the ER stress inducer thapsigargin (an inhibitor of sarco/endoplasmic reticulum Ca^2+^ ATPase) and the chemotherapeutic drug etoposide (an inhibitor of topoisomerase II). Both wild-type untransformed and transformed cells underwent cell death at comparable levels (Figure 1D). In contrast, Bak^−/−^Bax^−/−^ cells remained viable regardless of their transformation status, indicating that oncogenic transformation does not affect Bak/Bax-dependent apoptotic signaling in these models.

### TiO_2_ Nanoparticles Preferentially Induce Cell Death in Transformed Cells

**Figure 2:**
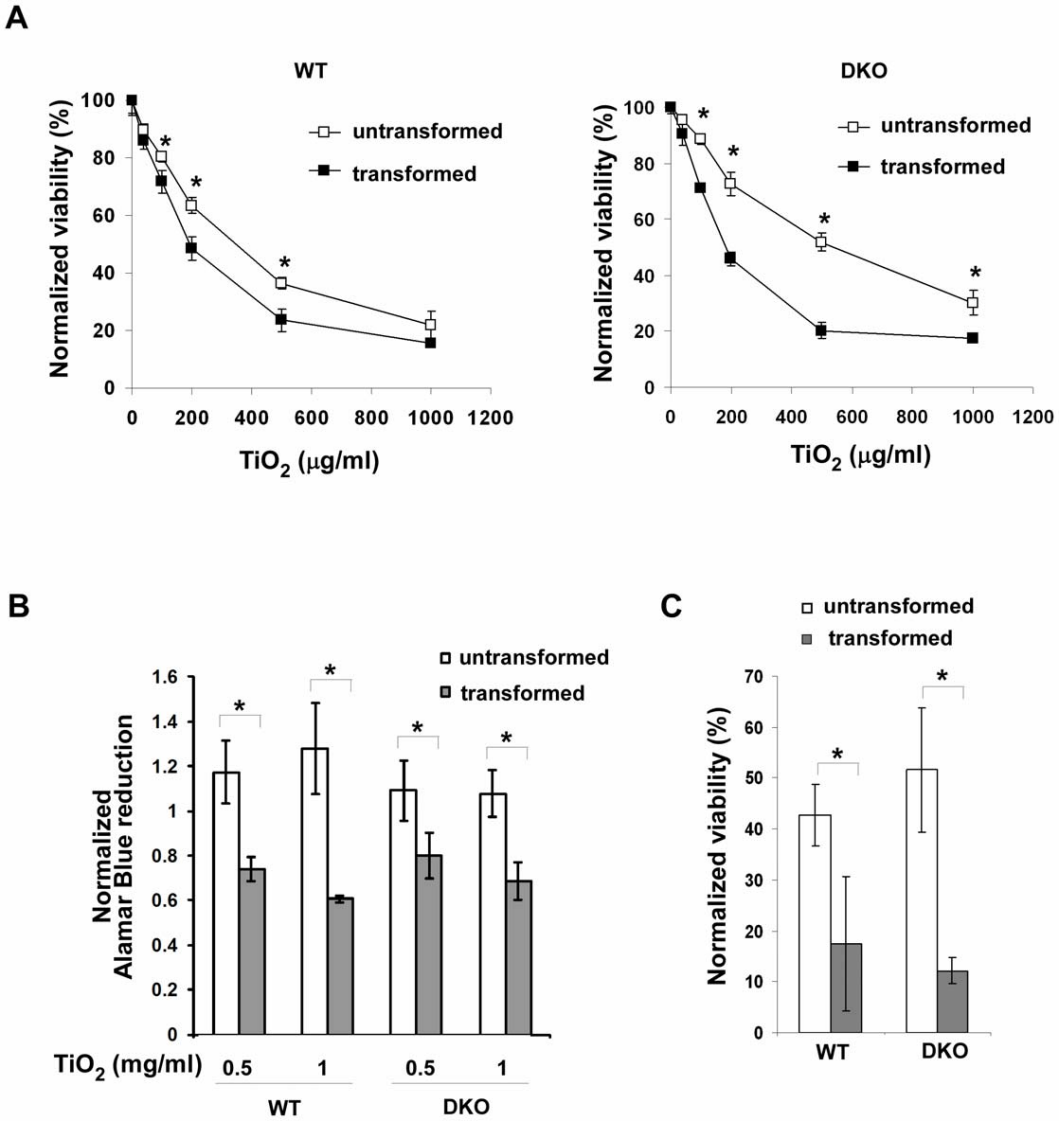
TiO_2_ nanoparticles preferentially induce cell death in transformed cells. (**A**) The indicated cell lines were treated with various concentrations of P25 TiO_2_ nanoparticles for 24 hours, and viability of cells was measured using TOTO-3 DNA dye exclusion method. Data represent mean±S.D. of three independent experiments. * p<0.05, Student’s unpaired t test. (**B**) Effects of TiO_2_ nanoparticles on cellular metabolic activities were determined by measuring Alamar Blue fluorescence. The indicated MEF cells were treated with 0.5 mg/ml or 1 mg/ml TiO_2_ nanoparticles for 24 hours. The cellular reducing activities of treated cells were normalized to that of corresponding untreated cell lines. Mean±S.D. of three independent experiments are shown. * p<0.05, Student’s unpaired t test. (**C**) Effects of TiO_2_ nanoparticles on long-term cell viability were determined by clonogenicity assay. The normalized cell survival was calculated by dividing the number of wells with viable treated cells with that of untreated cells. Data represent mean±S.D. of three independent experiments. * p<0.05, Student’s unpaired t test.

To systematically examine the effects of TiO_2_ nanoparticles on cells, two pairs of untransformed and transformed cells were treated with increasing concentrations of TiO_2_ nanoparticles (P25) for 24 hours. Cell viability was first measured using the cell viability indicator TOTO-3. Decreased viability was observed in all four cell lines tested in a dose-dependent fashion (Figure 2A). However, the viability of transformed wild-type and Bak^−/−^Bax^−/−^ cells was significantly lower than that of their untransformed counterparts at most tested TiO_2_ nanoparticle concentrations, suggesting that oncogenic transformation rendered the cells more sensitive to TiO_2_ nanoparticles. Interestingly, untransformed Bak^−/−^Bax^−/−^ cells were more resistant to the exposure of TiO_2_ nanoparticles than untransformed wild-type cells. However, Bak/Bax-dependent resistance in untransformed cells seems to be transient as the long term cytotoxicity of TiO_2_ nanoparticles was independent of Bak/Bax expression (Figure 2C).

To further confirm the differential cytotoxicity of TiO_2_ nanoparticles on transformed and nontransformed cells, we evaluated cellular health using Alamar Blue, whose reduction reflects cellular metabolic activities (Figure 2B). Upon 0.5 mg/ml or 1 mg/ml TiO_2_ nanoparticle treatment, metabolic activities of transformed wild-type and Bak^−/−^Bax^−/−^ cells were significantly lower than those of untransformed cells. Furthermore, metabolic activities of transformed wild-type and Bak^−/−^Bax^−/−^ cells were reduced at comparable levels, suggesting that the effects of TiO_2_ nanoparticles on cellular metabolic activities are independent of Bak/Bax. Overall, these results indicate that TiO_2_ nanoparticles are preferentially cytotoxic to transformed cells in a Bak/Bax-independent fashion.

While TOTO-3 exclusion and Alamar Blue reduction are effective measurements of short-term cytotoxicity, overall long term cell survival is better reflected by clonogenic assays. After 24 hours exposure to TiO_2_ nanoparticles, cells were plated into 384-well tissue culture plates at an average of one cell in each well, and the number of wells with cell colonies was counted two weeks later. While TiO_2_ nanoparticles decreased the long term viability of all cell lines tested, the viability of transformed cells was significantly lower than that of their untransformed counterparts (Figure 2C). Importantly, in all measures of cytotoxicity, the toxicity of TiO_2_ nanoparticles is independent of Bak/Bax-dependent apoptotic signaling.

### Oncogenic Transformation does not Affect Endocytosis

**Figure 3:**
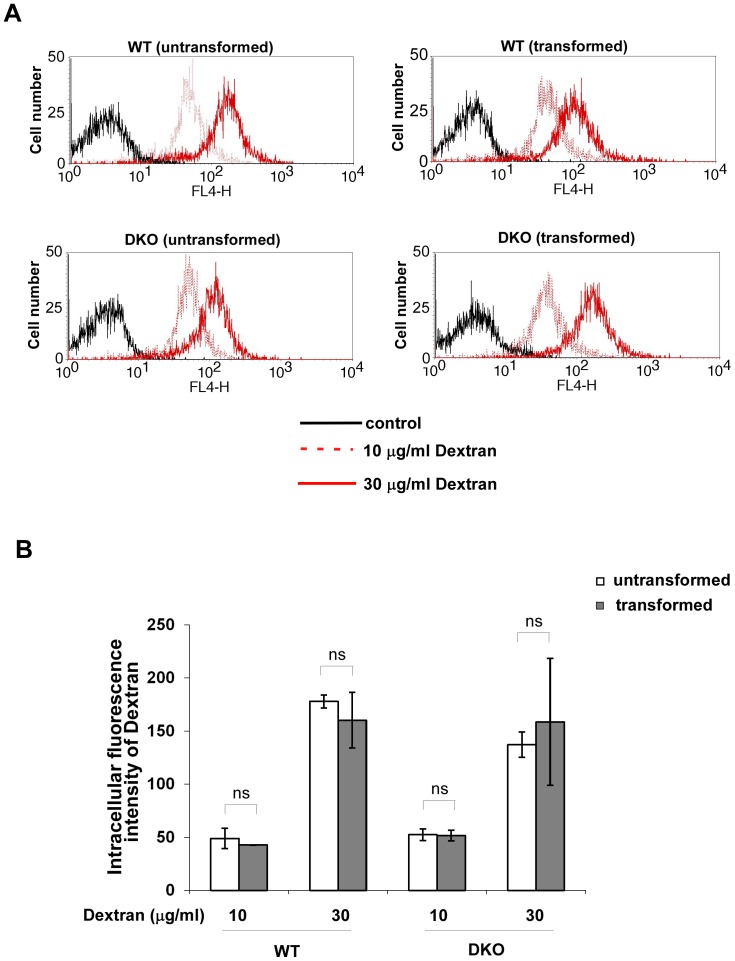
Oncogenic transformation does not affect fluid phase endocytosis. (**A**) Untransformed and transformed Wild-type or Bak^−/−^Bax^−/−^ MEF cells were incubated with 10 µg/ml or 30 µg/ml 10 kDa Dextran conjugated to Alexa Fluor 647 (Invitrogene). The uptake of Dextran into cells was measured by flow cytometry. (**B**) The intracellular levels of Dextran were measured as the intensities of Dextran fluorescence. Mean±S.D. of three independent experiments are shown. “ns” indicates no significance (P>0.05), Student’s unpaired t test.

One possible cause for the selective cytotoxicity of TiO_2_ nanoparticles on transformed cells could be that oncogenic transformation enhances endocytosis. To test this possibility, we used 10 KDa dextran-conjugated Alexa Fluor 647 to examine fluid phase endocytosis in both transformed and untransformed cells (Figure 3). At the concentrations of 10 or 30 µg/ml, dextran uptake of transformed cells was comparable to that of their untransformed counterparts. These results indicate that the increased cytotoxicity of TiO_2_ nanoparticles on transformed cells is probably not due to an increase in endocytosis. However, we must point out that endocytotic uptake of fluids and particles may not be exactly the same so we carried out similar estimates of nanoparticle uptake as described below.

### Uptake of TiO_2_ Nanoparticles is Similar in Transformed and Untransformed Cells

**Figure 4:**
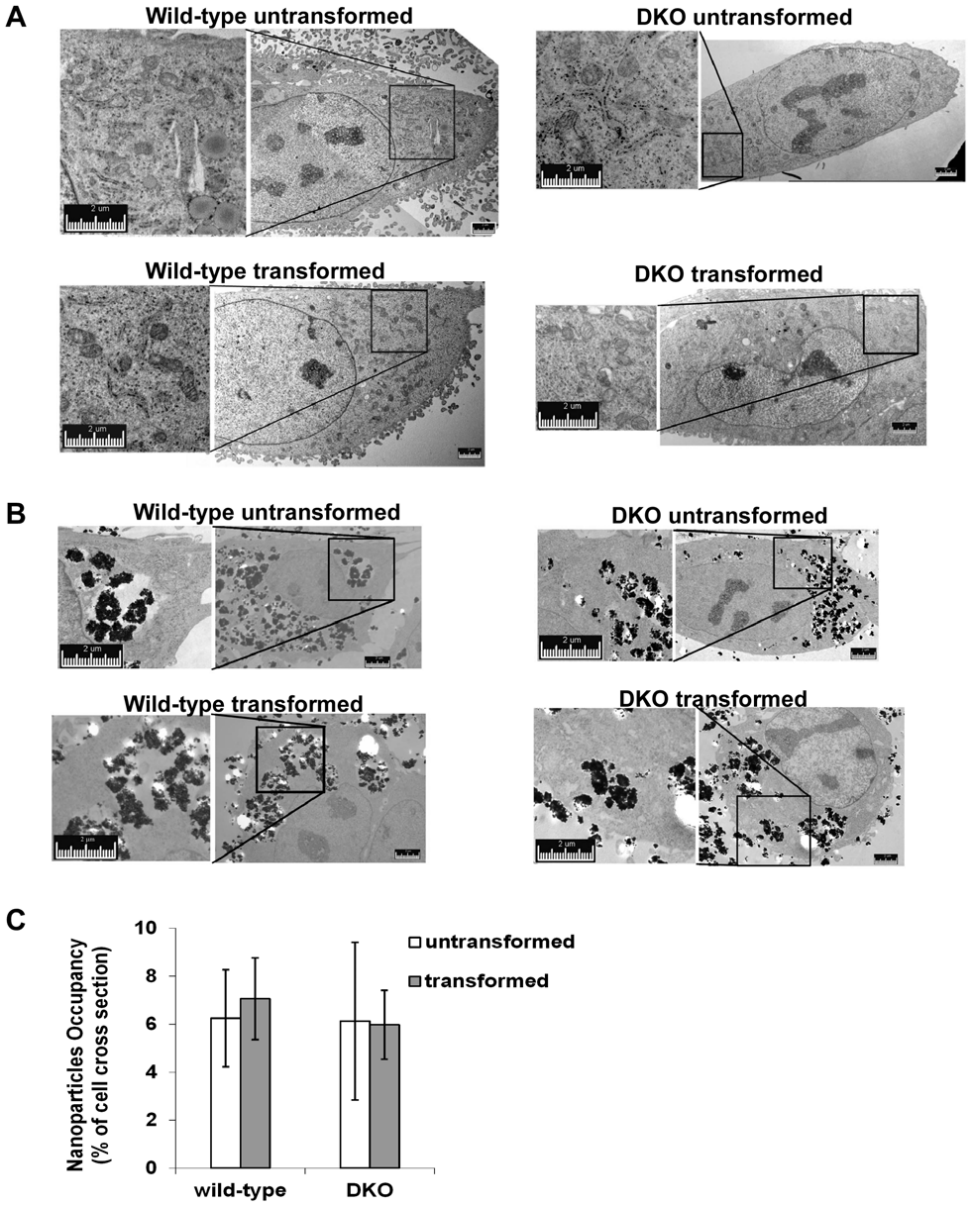
The abundance of intracellular TiO_2_ nanoparticles is not influenced by oncogenic transformation. The indicated MEF cells were cultured with or without 0.5 mg/ml TiO_2_ nanoparticles for 24 hours. The subcellular structures of control and treated cells were examined by transmission electron microscopy (TEM). (**A**) In the absence of nanoparticles, the indicated MEF cells exhibit comparable subcellular structures. Representative cross section images of the indicated cells are shown. The left panels are the enlargement of the corresponding inlets in the panels on the right. The large electron microscopic images were generated by stitching several images of smaller regions together. (**B**) Localization and abundance of intracellular TiO_2_ nanoparticles were determined by TEM. Representative TEM images of cross sections of the indicated cells show the intracellular presence of TiO_2_ nanoparticles. The large electron microscopic images were generated by stitching several images of smaller regions together. (**C**) The abundance of TiO_2_ nanoparticles in the treated cells was evaluated as the percentage of the total cellular area occupied by nanoparticles in cross sections of cells. Data represent mean±S.D. of at least 7 randomly selected independent cross sections.

Electron microscopy experiments were carried out to examine the intracellular distribution and abundance of TiO_2_ nanoparticles in cells. In untreated control cells, subcellular organelles, such as mitochondria and endoplasmic reticulum, were easily observed by transmission electron microscopy (Figure 4A). After 24 hour exposure of cells to 0.5 mg/ml TiO_2_ nanoparticles, clusters of TiO_2_ nanoparticles inside cells were sequestered within vacuoles, likely representing endosomes and lysosomes (Figure 4B). Some of intracellular nanoparticles were localized in the cytoplasm, perhaps as a result of lysosomal membrane rupture. There was no significant difference in the intracellular abundance of TiO_2_ nanoparticles between transformed and untransformed cells, suggesting that intracellular TiO_2_ nanoparticle abundance is unlikely the cause of selective cytotoxicity of TiO_2_ nanoparticles on transformed cells (Figure 4C).

### Intracellular TiO_2_ Nanoparticles Colocalize with Lysosomes

**Figure 5:**
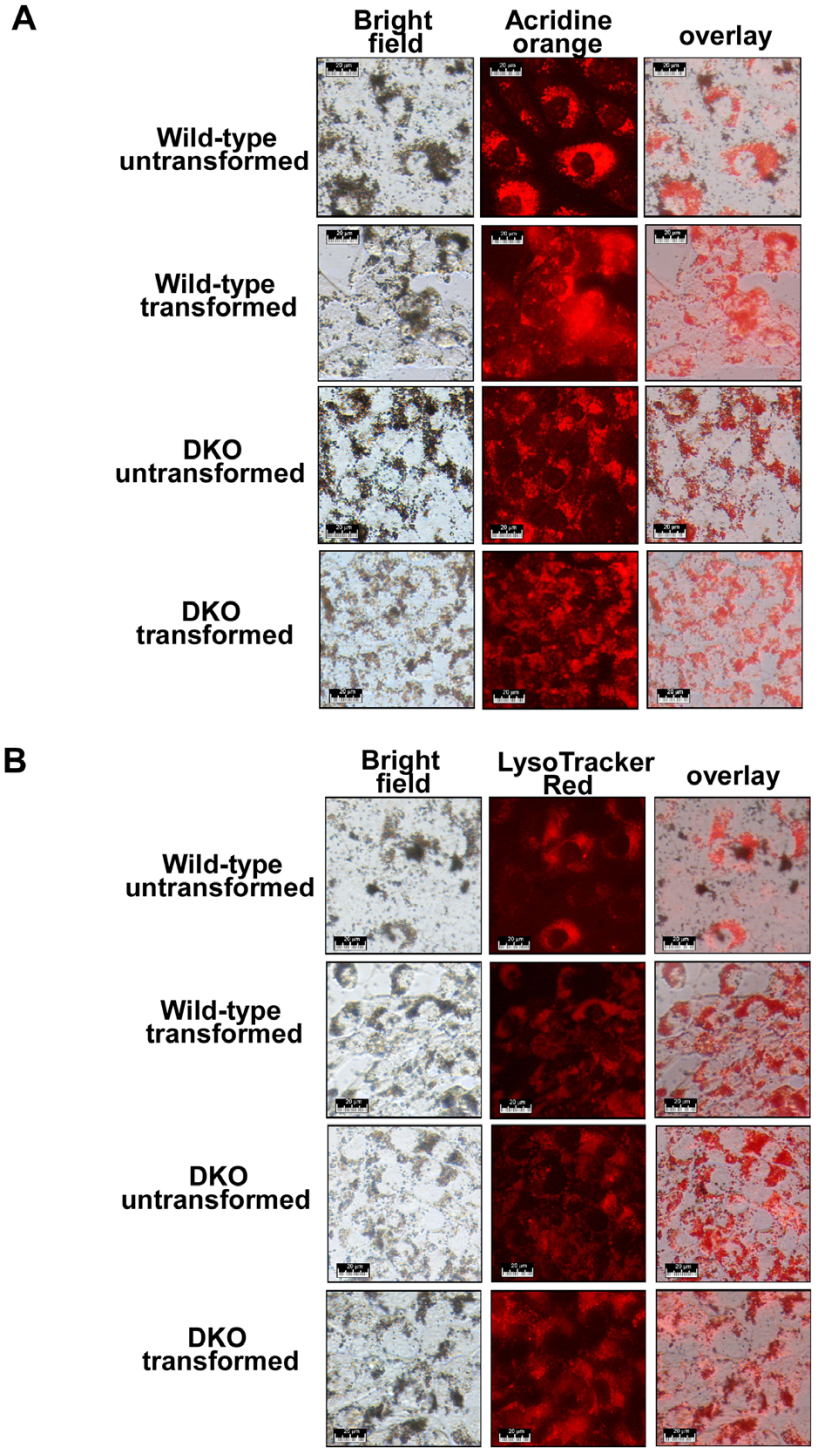
Intracellular nanoparticles colocalize with lysosomes. The indicated cell lines were first cultured with 0.1 mg/ml nanoparticles for 24 hours, then incubated with 2 µM acridine orange (A) or 50 nM LysoTracker Red (B). Bright field and fluorescence images were acquired through a fluorescence microscope. Representative images are shown. While lysosomes labeled with acridine orange or LysoTracker Red are shown in red (the middle panels), TiO_2_ nanoparticles inside cells are observed as dark-colored aggregates (the left panels).

To further determine whether intracellular nanoparticles are localized in lysosomes, we used two approaches to label lysosomes. Acridine orange is a traditional reagent labeling lysosomes, which efficiently labels lysosomes from sub micromolar to low micromolar range [34]. LysoTracker Red is a recently developed reagent specifically labeling lysosomes at very low concentrations, which identifies lysosomes in the 10–70 nM range. Both reagents label acidic lysosomes generating red fluorescence under a fluorescence microscope. Upon exposure to TiO_2_ nanoparticles, dark-colored aggregates of TiO_2_ nanoparticles inside cells were observed under a bright field microscope (Figure 5). Lysosomal labeling by both acridine orange and LysoTracker Red exhibited a perinuclear distribution (Figure 5). Importantly, intracellular nanoparticles were largely colocalized with lysosomal labeling by either acridine orange or LysoTracker Red in all the four cell lines tested. These results are consistent with previous studies in other cell lines [10].

### Oncogenic Transformation Enhances Lysosomal Activities

**Figure 6:**
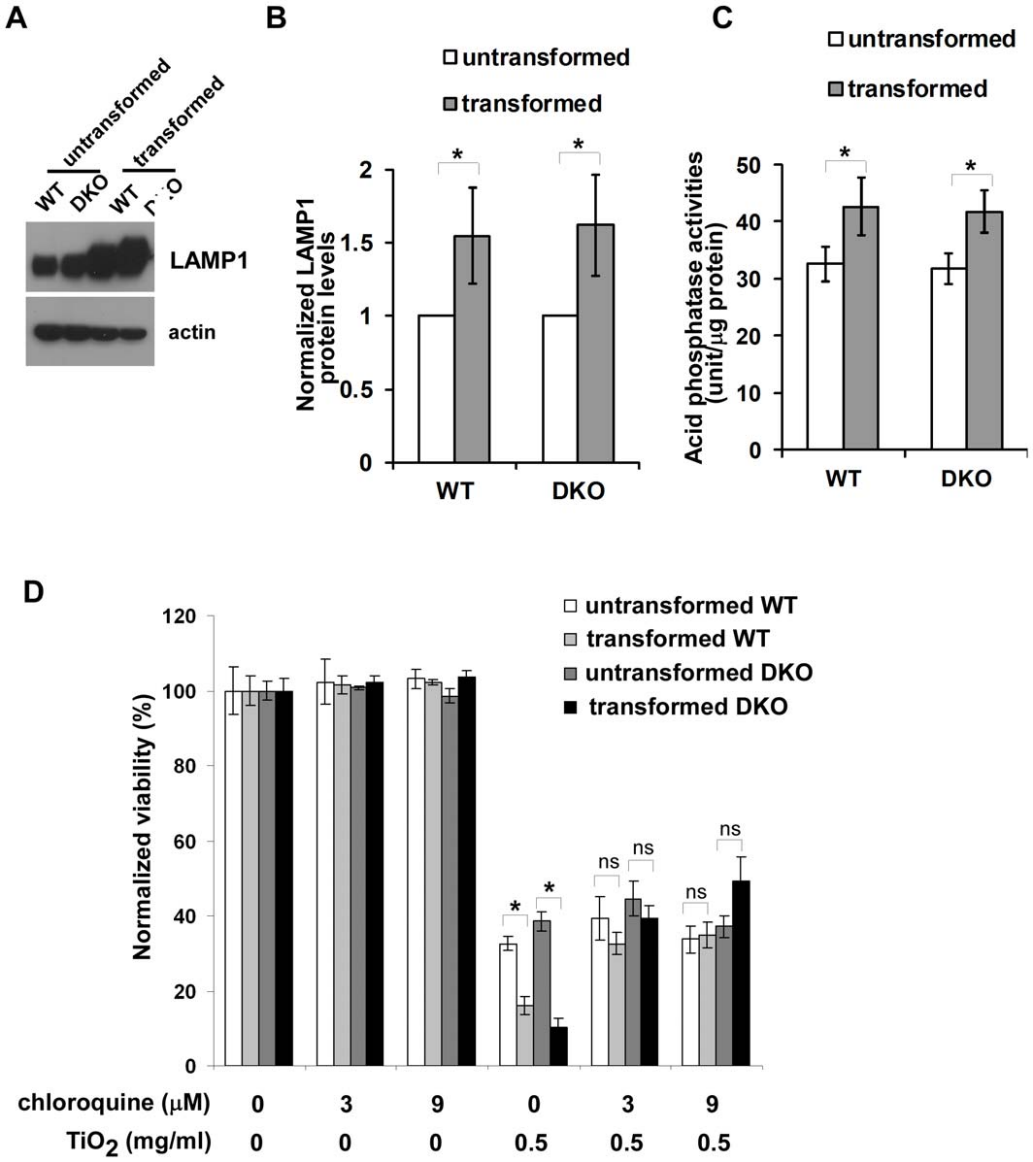
Selective cytotoxicity of TiO_2_ nanoparticles is related to an oncogenic transformation-induced increase of lysosomal activities. (**A**) The expression levels of lysosomal protein LAMP1 in the indicated cells were determined by western blot. (**B**) The expression levels of LAMP1 are higher in transformed cells than their untransformed counterparts. The intensities of LAMP1 and actin shown in (A) were quantified using ImageJ software (NIH). The relative LAMP1 levels were calculated with the intensity of LMAP1 normalized to that of actin in the same sample. Data represent mean±S.D. of three independent experiments. * p<0.05, Student’s unpaired t test. (**C**) The enzymatic activities of lysosomal acid phosphatase were measured. Mean±S.D. of three independent experiments are shown. “*” indicates P<0.05, Student’s unpaired t test. (**D**) Chloroquine alleviates death of transformed cells induced by TiO_2_ nanoparticles. The indicated cells were cultured in the absence or presence of chloroquine or TiO_2_ nanoparticles. Cell viability was measured 24 hour after the treatments using TOTO-3 DNA dye exclusion approach. Data represent mean±S.D. of three independent experiments. “*” indicates P<0.05; “ns” indicates no significance (P>0.05), Student’s unpaired t test. The viabilities of TiO_2_ nanoparticle-treated transformed cell lines in the presence of chloroquine are significantly higher than those in the absence of chloroquine (p<0.05, Student’s unpaired t test), whereas the differences in the viabilities of untransformed cells with and without chloroquine exposure are not statistically significant (Student’s unpaired t test).

It has been reported that oncogenic transformation enhances the intracellular lysosomal system, which is associated with tumor invasion and metastasis [35], [36]. We explored the possibility that oncogenic transformation mediated by K-Ras and E1A could also increase lysosomal activities using two different approaches. First, we examined the influence of oncogenic transformation on expression levels of the lysosomal marker lysosomal-associated membrane protein 1 (LAMP1), a glycosylated integral membrane protein that is highly enriched in late endosomes and lysosomes [37]. The expression levels in both transformed wild-type and Bak^−/−^Bax^−/−^ MEF cells were higher than those in their untransformed counterparts (Figure 6A). Acid phosphatases are a class of lysosomal enzymes that hydrolyze phosphomonoesters [38]. As activities of acid phosphatase are important for digestive functions of lysosomes, we examined whether oncogenic transformation increases acid phosphatase activities. As shown in Figure 6C, acid phosphatase activities were significantly higher in transformed cells than those in the corresponding untransformed cells. Overall, these data suggest that oncogenic transformation mediated by Ras and E1A enhances lysosomal activities.

### Chloroquine, a Lysosomotropic Base, Reduces TiO_2_ Nanoparticle Cytotoxicity

Next, we investigated whether enhanced lysosomal activities in transformed cells may contribute to increased cytotoxicity induced by TiO_2_ nanoparticles. As a weak base, the anti-malarial medicine chloroquine has been shown to concentrate in lysosomes and inhibit lysosomal activities [39]. Chloroquine was added to the cell cultures at the same time as TiO_2_ nanoparticles, and the viability was analyzed using flow cytometry 24 hours later (Figure 6D). Viability of all four MEF cell lines was not affected by 3 µM or 9 µM chloroquine alone. However, chloroquine treatment reduced the TiO_2_ nanoparticle cytotoxicity on transformed cells but not untransformed cells, suggesting that lysosomal activities are important for enhanced cytotoxicity of nanoparticles on transformed cells.

## Discussion

Although the cytotoxicity of TiO_2_ nanoparticles has been investigated extensively, precise mechanisms through which TiO_2_ nanoparticles induce cell death are mostly unclear. To explore the role of oncogenic transformation in modulating cellular responses to TiO_2_ nanoparticles, we study cells transformed by oncogenes K-Ras and E1A and their untransformed isogenic counterparts. In this paper, we find that transformed cells are more sensitive to TiO_2_ nanoparticles than the corresponding untransformed cells by several different approaches. Importantly, the cytotoxic activities of TiO_2_ nanoparticles are not involved in Bak/Bax-mediated major apoptotic signaling pathways. While oncogenic transformation does not appear to affect the entry of TiO_2_ nanoparticles into cells, transformation-induced lysosomal activity increase might contribute to the selective cytotoxicity of TiO_2_ nanoparticles. Together, our studies provide molecular evidence that TiO_2_ nanoparticles preferentially cause tumor cell death through a lysosome-mediated pathway.

Bak and Bax are redundant pro-apoptotic regulators of apoptotic responses. Together they control the majority of apoptotic machinery except the extrinsic cell death pathway in thymocytes [27]. Cell death in the absence of Bak and Bax is largely associated with other forms of cell death, particularly necrosis [40]. Both apoptosis and necrosis has been reported to occur in cells treated with TiO_2_ nanoparticles, and the prevalence of a particular cell death paradigm depends not only on particle properties but on cell types as well [10]. Our studies show that TiO_2_ nanoparticle toxicity is independent of Bak/Bax-mediated apoptotic signaling in measurement of both acute and long term of cytotoxicity, supporting the notion that TiO_2_ nanoparticles cause non-apoptotic cell death. An increase of reactive oxygen specie production has been observed in cells exposed to TiO_2_ nanoparticles [18]–[21]. In addition, severe oxidative stress is associated with lysosomal membrane permeabilization and subsequent necrosis, which is controlled by complicated signaling pathways [41], [42]. Given the evidence of lysosomal involvement in TiO_2_ nanoparticle cytotoxicity, it is conceivable that necrosis might account for the majority cell death observed in our studies.

Uptake of insoluble particles such as calcium oxalate is thought to promote cell death by causing cytosolic membrane instability [43]. This is unlikely to explain the differential toxicity of TiO_2_ nanoparticles to transformed cells inasmuch as the uptake of these nanoparticles in both cell types is equivalent. In support of this, close examination of electronic microscopic images of different cells incubated with TiO_2_ nanoparticles fails to reveal differences in cytoplasmic membrane integrity between transformed and untransformed cells (Figure 4). In our studies, TiO_2_ nanoparticles appear to enter cells via endocytosis, and intracellular TiO_2_ nanoparticles are largely localized within the lysosomal apparatus (Figures 4 and 5). Thus, the differential cytotoxicity of TiO_2_ nanoparticles suggests that the difference in lysosomal function between transformed cells and the corresponding untransformed cells might be involved in the selective cytotoxicity of TiO_2_ nanoparticles. Indeed, we find that lysosomal abundance and lysosomal enzymatic activities (e.g. acid phosphatase) appear to be enhanced by oncogenic transformation. This is consistent with emerging evidence that transformation leads to dramatic changes in lysosomes, including increases in lysosomal size, protease activity, and protease secretion, which are correlated with invasive growth and angiogenesis of tumors [35], [44]. Importantly, recent findings demonstrate that these changes in the lysosomal compartment sensitize tumor cells to lysosomal membrane rupture, resulting in subsequent cell death, including necrosis. Conceivably, increased lysosomal activities cause transformed cells to be more sensitive to TiO_2_ nanoparticles by inducing higher levels of lysosomal membrane permeabilization. As a weak base, chloroquine has been shown to increase pH in acidic organelles (e.g. lysosomes) to inhibit the transport of endocytosed particles from endocytic vesicles to lysosomes without significantly affecting the initial uptake of exogenous metal particles into the cells [45], [46]. It is unlikely that the ability of chloroquine to reduce TiO_2_ nanoparticle toxicity in transformed cells can be attributed to its influence on TiO_2_ nanoparticle uptake.

As the difference in processing TiO_2_ nanoparticles inside lysosomes likely contributes to their selective cytotoxicity, the toxicity might be due to their pro-oxidant effects within lysosomes. Ionic titanium is a known Fenton-type reagent which fosters the generation of hydroxyl radicals [47], [48], and it is possible that TiO_2_ nanoparticle cytotoxicity involves the conversion of TiO_2_ nanoparticles to ionic titanium in lysosomes. Lysosomes are acidic organelles filled with acid hydrolases, and the pH within the lysosomal compartment is estimated at about 4.5 [49]. In addition, lysosomes are also rich in reducing agents such as cysteine [50]. Thus, the reduction of TiO_2_ nanoparticles to ionic titanium could occur in the acidic and reducing environment of lysosomes. As a result, enhanced lysosomal capacities in transformed cells could promote the conversion of metallic to ionic titanium, leading to enhanced generation of reactive oxygen species and subsequent cellular damage. In support of this, the lysosomotropic base chloroquine reduces TiO_2_ nanoparticle cytotoxicity in our studies (Figure 6D). Whether the conversion of TiO_2_ to ionic titanium is responsible for the observed cytotoxicity merits further investigation.

Oncogenic transformation of fibroblasts induces cell morphological changes from flat to round shapes, typical of malignant tumor cells [51]. Similar changes in morphology were also observed in both wild-type and Bak^−/−^Bax^−/−^ MEFs transformed by K-Ras and E1A. In a previous report, the morphology of human monocyte-derived macrophages is altered by contact with different substrata [55]. While inflammation-related reactive oxygen intermediate (ROI) production is suppressed in macrophages with flat morphology, cells with round shape generate abundant cytotoxic ROI. Thus, it is conceivable that the difference in susceptibility to TiO_2_ nanoparticles between transformed and untransformed cells reported here might be attributed to oncogenic transformation-mediated alteration of cell architecture.

In summary, our studies provide evidence that the selective cytotoxicity of TiO_2_ nanoparticles in transformed cells might be involved in a lysosome-mediated cell death. This preferential killing of transformed cells by TiO_2_ nanoparticles might reflect an Achilles heel of cancer cells which could be exploited therapeutically.

## Methods

### Reagents and Cell Culture

Titanium dioxide (TiO_2_) nanoparticles with nominal size 25 nm (P25) were kindly provided by Dr. Liping Tang (University of Texas, Arlington). Thapsigargin and chloroquine were obtained from Sigma Aldrich (St. Louis, MO). Etoposide was purchased from Enzo (Plymouth meeting, PA). 10 KDa Dextran conjugated to Alexa Fluor 647 was purchased from Invitrogen (Carlsbad, CA). Antibodies used for western blot analysis were anti-Mcl-1 mAb (Epitomics; Burlingame, CA), anti-Bcl-X_S/L_ S-18 pAb (Santa Cruz; Santa Cruz, CA), anti-mouse Bcl-2 mAb (Santa Cruz), anti-β-actin mAb (Sigma), anti-Bak, NT pAb (Upstate; Lake Placid, NY), anti-Bax pAb (Santa Cruz), and anti-LAMP1 pAb (Santa Cruz, CA). Untransformed and transformed MEF cells were kindly provided by Dr. Wei-Xing Zong [52] and were cultured in DMEM/high glucose medium (Mediatech; Manassas, VA) containing 10% (v/v) fetal bovine serum (Gemini, West Sacramento, CA), 2 mM glutamine (Mediatech), 100 U/ml penicillin and 100 µg/ml streptomycin (Mediatech). Cells were grown in a humidified 95/5% air/CO_2_ incubator at 37°C.

### Western Blot Analysis

One million MEF cells were collected by centrifugation, and cell pellets were resuspended in 1X LDS loading dye (Invitrogen). Cell lysates were sonicated for 5 seconds at 10% amplitude (Sonic Dismembrator, Model 500, Fisher Scientific; Hampton, NH) before heating at 100°C for 5 minutes. Lysates corresponding to 1×10^5^ cells were electrophoresed in 4–12% Bis-Tris gels (Invitrogen) and transferred to PVDF (Millipore; Billerica, MA) for incubation with the appropriate primary and secondary antibodies. Proteins were detected using ECL western blotting substrate (Pierce; Rockford, IL). The intensity of proteins was quantified using ImageJ software (version 1.42q, NIH; Bethesda, MA).

### Soft Agar Colony Formation Assay

2 ml of 0.6% agar containing 10,000 exponentially growing cells for each well was poured into a 6-well tissue culture plate. 2 ml DMEM growth medium was added on top of the solidified agar and the plates were incubated in a 37°C in a tissue culture incubator. Medium was replaced with fresh growth medium every 4 days. After 10 day incubation, the liquid cell culture medium was removed, and 1 ml of 12 mM 3-(4,5-Dimethylthiazol-2-yl)-2,5-diphenyltetrazolium bromide (MTT, Sigma) was added to the plates. Following 15 minutes incubation, the images of the plates were photographed, and the number of colonies on randomly selected images was counted.

### Treating Cells with TiO_2_ Nanoparticles

MEF cells were grown for 48 hours in a 37°C incubator prior to TiO_2_ nanoparticle treatment. Immediately before addition to cells, medium containing TiO_2_ nanoparticles (P25) was prepared by adding TiO_2_ nanoparticles to fresh growth medium at the indicated concentrations and sonicated with three 10-second pulses at 20% power (Sonic Dismembrator model 500, Fisher Scientific). The growth medium was replaced with the medium containing TiO_2_ nanoparticles.

### Cell Viability Analysis by Flow Cytometry

Cells were treated with Trypsin-EDTA (Mediatech) and harvested. DNA dye TOTO-3 (Invitrogen) was added at the final concentration of 1 µg/ml. Cell viability was measured by TOTO-3 exclusion using flow cytometry (FACSCalibur, Becton Dickinson; Franklin Lakes, NJ) as described previously [53]. Data were normalized to the viability of untreated cells.

### Measuring Cytotoxicity of TiO_2_ Nanoparticles by Dye Reduction

Cell were plated in 96-well tissue culture plates for 48 hours, and incubated with the growth medium containing the indicated concentrations of TiO_2_ nanoparticles. Cell metabolic activities were determined by measuring Alamar Blue (AbD Serotec, Raleigh, NC) reduction as described previously [54]. Briefly, following TiO_2_ nanoparticle exposure, cells were incubated with fresh growth medium containing 10% Alamar Blue (v/v). Alamar Blue reduction was determined by measuring fluorescence with 560 nm excitation and 590 nm emission using a SpectraMAX spectrofluorometric plate reader (GeminiEM, Molecular Devices Corp.; Sunnyvale, CA). Measurements were carried out 1 hour after Alamar Blue addition for two hours at 30 minute interval, and the linear region of the response curve was used for calculation.

### Clonogenicity Assay

Cells with or without TiO_2_ nanoparticle treatment were collected, and cell concentrations were determined. Cells were diluted to a concentration of 33 cells per ml, and a 30 µl aliquot was added into each well of 384-well tissue culture plates with one cell in each well on average. The plates were incubated for 14 days in a 37°C incubator. Cells were stained with 2% crystal violet for 30 minutes at room temperature, and washed extensively with distilled H_2_O. The number of wells containing cell colonies was counted under a microscope.

### Measuring TiO_2_ Nanoparticle Cellular Occupancy Using Electron Microscopy

Cells were plated on ACLAR film disks (Jed Pella; Redding, CA) in 12-well tissue culture plates with 20,000 cells in each well. After incubation for 48 hours, TiO_2_ nanoparticles were loaded onto cells by replacing the growth medium with medium containing the indicated concentrations of nanoparticles. 24 hours later, the treated cells were fixed using 3% glutaraldehyde in cacodylate buffer (0.1 M cacodylate in 1X PBS) at room temperature for 3 hours. Cells were then washed twice for 5 minutes each time with 0.1M cacodylate buffer. The cells on the ACLAR disks were then post fixed using 1% osmium tetroxide in cacodylate buffer, dehydrated in graded ethanol (60%, 90% and 100%), and embedded in LX-112 epoxy resin (Ladd Research; Williston, VT). Ultra-thin 80 nm sections were cut, mounted on uncoated copper grids, and stained with lead citrate and saturated aqueous uranyl acetate. Images were examined with a Philips CM12 transmission electron microscope (Philips, Andover, MA) at 80kV. TiO_2_ nanoparticle cellular occupancy was determined by the area ratio in the traverse cellular sections. First, non-cellular areas on each electron microscopic image were manually defined and removed using ImageJ software (version 1.43, NIH). Appropriate brightness threshold was set to select the areas containing dark TiO_2_ nanoparticles. The nanoparticle occupancy was calculated as the ratio of the nanoparticle-containing area versus the total cellular area.

### Measuring Endocytosis Capacity of Cells

10,000 of the indicated cells were plated in a 48-well tissue culture plate. 48 hours later, tissue culture medium containing 0, 10 or 30 µg/ml 10 KDa Dextran conjugated to Alexa Fluor 647 (Invitrogen) was added to cells. Following 90 minutes incubation in a 37°C incubator, cells were harvested. The fluorescence intensity of Alexa Fluor 647-conjugated Dextran was determined using flow cytometry (FL4-H, FACSCalibur).

### Identification of Nanoparticle Colocalization with Lysosomes

The indicated cell lines were plated in a 48-well tissue culture plate and incubated at 37°C for 48 hours. 2 µM acridine orange or 50 nM LysoTracker Red was added to the growth medium. After 30 minutes incubation, bright field and fluorescent images were taken using a Nikon TS100 microscope with a Nikon DSL1 digital camera. The peak transmission of the excitation filter was at 540 nm, while that of the emission filter was at 580 nm.

### Acid Phosphatase Assays

Cells were collected and lysed in the buffer containing 1% Triton X-100, 0.1% SDS, and protease inhibitors (Complete, Roche, Indianapolis, IN). Insoluble cell debris was removed by centrifugation, and protein concentration was determined by the BCA Protein Assay (Thermo Fisher Scientific, Rockford, IL). Acid phosphatase enzymatic activities of lysate containing 20 µg protein were measured using the Acid Phosphatase Assay Kit (Sigma Aldrich) as described by manufacturer. One unit of acid phosphatase will hydrolyse 1 pmole of 4-nitrophenyl phosphate per minute at Ph 4.8 at 37°C.
